# Using an Attachment System with PEEK Matrices for Single-Implant Overdentures: In Vitro Retention Force

**DOI:** 10.3390/jcm12062159

**Published:** 2023-03-10

**Authors:** Ioana Fugariu, Raphael Freitas de Souza, Eduardo Rosas, Eduardo Borie

**Affiliations:** 1 Faculty of Dental Medicine and Oral Health Sciences, McGill University, Montreal, QC H3A 1G1, Canada; 2 Master in Dental Sciences Program, Universidad de La Frontera, Temuco 4811230, Chile; 3 CICO Research Centre, Adults Integral Dentistry Department, Universidad de La Frontera, Temuco 4811230, Chile

**Keywords:** complete denture, denture attachment, implant-supported dental prosthesis, prosthesis failure

## Abstract

Single-implant overdentures (SIOs) represent a major biomechanical challenge in terms of prosthetic retention. The Novaloc attachment system has the potential to overcome those challenges when used for SIOs, due to the use of PEEK matrices. This study compared the retentive force of the Novaloc attachment to the traditional Locator system, before and after cyclic insertion–removal cycles. Three Novaloc matrices (white, yellow, and green, corresponding to low, medium, and high retention, respectively) and Locator (medium retention) were tested, totalling four groups. Retentive force was measured using an Imada force gauge before and after 1095 insertion–removal cycles, corresponding to a year of SIO wearing. Retention was tested with the implants angulated at 0, 10, and 20°. Data for the different groups, angles, and cycling periods were tested via linear regression analysis and two-way ANOVA (α = 0.05). Although the Locator system yielded higher retention forces in general, it lost a much higher percentage of retention with cycling. This trend was similar with the three angles, with forces being inversely proportional to the implant angulation. The authors conclude that Novaloc may provide more reliable retention for SIOs due to their higher resistance to insertion–removal cycling.

## 1. Introduction

Edentulism is still a common condition worldwide, and it represents a major burden on patients’ well-being [1]. Removable complete dentures are a common treatment for edentulism, even if the masticatory performance with this appliance is roughly one third of dentate individuals [2]. The most cost-effective way to overcome those functional limitations is the mandibular overdenture, retained by implants in the anterior mandible [3,4]. Implant retention is often provided by stud or bar-type attachments, both composed of a rigid structure screwed into the implants and a flexible matrix embedded into the overdenture [5]. When the denture is seated, the matrix contours undercuts around the screwed structure and must be elastically deformed for denture removal. Regardless of the type and number of attachments, overdentures perform significantly better than conventional dentures both from the patient and clinician perspectives [6].

The performance of implant-retained mandibular dentures is often excellent, even with a reduced number of implants [3]. International consensuses recommend the two-implant mandibular overdenture as a standard of care for edentulous patients compared to other alternatives [4,7]. Despite the advantages offered by this model, fear of oral surgery, cost, systemic diseases that restrict procedure times, and narrow ridges might limit the placement of two implants over a single implant in the middle of the ridge [8]. Contrary to previous beliefs, evidence suggests that the retention of a mandibular overdenture by one implant (or single-implant overdenture—SIO) positioned in the midline of the mandible is viable. Compared to two-implant overdentures, it has potential for lower morbidity and seems to have similar longevity [9]. In reality, a single implant concentrates tension favorably, without the harmful loading of the abutment, the peri-implant bone, or the edentulous ridge [10].

Despite the potential benefits offered by SIO, its reliance on a single attachment outlines the importance of the regular maintenance of this attachment. Implant attachments undergo repetitive forces in use, being deformed several hundreds of times daily [11]. The continuous removal and insertion of overdentures may also wear attachments, leading to reduced retentive force [12,13]. This results in a need for routine reactivation or change in the retentive components, which may become inefficient/costly for the intended population (e.g., elderly patients). This has been evidenced in the most used attachment in North America (the Locator system) [14], which tends to lose retention with few months of normal use due to the considerable deformation/wear of its nylon matrices [15,16]. This results in frequent changes of matrices, amongst other signs of mechanical failure [17]. Alternative biomaterials have the potential to mitigate the limitations of implant attachments, e.g., stronger polymers may reduce maintenance needs. Polyetheretherketone (PEEK) is one of the best alternatives to nylon given its superior strength [18] and resistance to creep [19]. PEEK matrices have the potential to improve an already efficient treatment approach for edentulism by tackling its weakest point [20]. A newer alternative to the mentioned traditional attachments that utilize PEEK matrices is the Novaloc system [21]. In this system, matrices are made of PEEK, which has higher wear resistance than the traditionally used nylon [22]. Abutments are coated by amorphous diamond-like carbon (ADLC) to reduce roughness and thus render retention more durable. A previous study has shown promising data on the retentive properties of Novaloc after cyclic insertion–removal [22]. However, there are no data on the Novaloc PEEK matrix with different levels of retention, nor any study specifically aimed at its application in SIOs.

To address this gap in knowledge, this in vitro study aims to understand the retentive capacity of the Novaloc PEEK attachments as an alternative to Locator nylon attachments for SIOs. These results are expected to provide more reliable clinical recommendations to treat edentulous patients.

## 2. Materials and Methods

This in vitro study quantified the retentive force of various Novaloc attachments and a Locator attachment before and after insertion–removal cycling, as expected with regular SIO wear.

### 2.1. Sample Preparation

**Figure 1:**
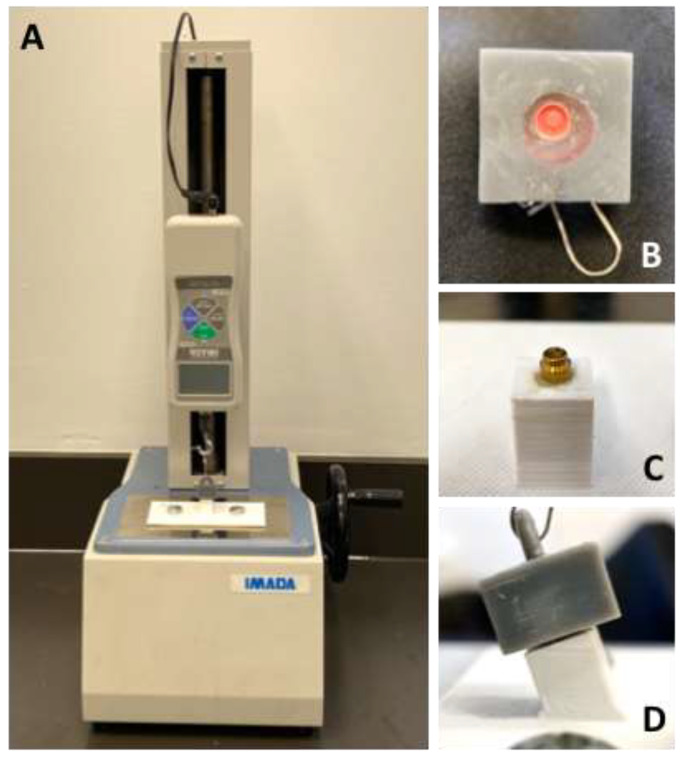
Force measurement gauge used in this study. (**A**) Assembled Imada DS2-500N equipment with (**B**) block with plastic matrix (gray plastic) and (**C**) base containing implant analog and abutment (white plastic). The example shows a Locator abutment with the respective medium/pink matrix. (**D**) Matrix attached to the abutment, in the block with 20°.

Firstly, the test samples were prepared by placing the following attachments over a single implant analog, forming 4 groups (n = 10 each). Three groups comprised Novaloc ADLC-coated abutments combined with each of the three different PEEK matrices—(1) light (white), (2) medium (yellow), and (3) strong (green) retention. The 4th group (control) used Locator TiN-coated abutments with pink (medium) retentive components. Three DLP 3D-printed plastic blocks were utilized to embed the implant analogs and matrices. In a clinical setting, a variety of factors (e.g., lack of bone or the shifting of teeth) may not allow for implant placement according to a straight path of insertion. Thus, three blocks were printed with platform inclinations angles of 0, 10, and 20°. Chairside hard reline (GC Reline, GC America, Alsip, IL, USA) was used to fasten the implant analogs to the blocks. Another single printed plastic block was printed to house the attachment matrix and housing. The blocks were attached to an Imada DS2-500N digital force measurement gauge (Imada, Inc., Northbrook, IL, USA; Figure 1) [15,23], which recorded the peak retention forces (Newtons) before and after a number of insertion–removal cycles corresponding to 1 week and 1, 3, 6, and 12 months (total: 1, 080 cycles, considering that a patient would remove their overdenture 3 times daily).

### 2.2. Cycling

The cycling of attachments was carried out manually by the experimenter. The top block (containing the attachment matrix and housing) was attached and detached from the bottom block (containing the implant analog and abutment) to stimulate the removal and insertion of an SIO in a patient. Taking into consideration the removal of the denture by a patient at night in addition to maintenance between meals (e.g., 3 meals a day), the removal–insertion cycling was set to 3 times daily. The retention of the attachments was measured using the force gauge machine [15,23] prior to any cycling (T1). To obtain this measurement, the top block was fastened to the machine and was dissociated from the bottom block by pulling the machine’s lever. This insertion–removal movement counted as part of the cycling process; as such, it was subtracted from each round of insertion–removal cycle (e.g., T2, 3, 4, 5, 6 and 7). To minimize the imprecision associated with the manual pulling of the lever, the readings were recorded in triplicate.

After the initial reading of each attachment (as mentioned above), each attachment was cycled to replicate the retention loss at: 1 week (T2 = 3 insertion-removal cycles/day × 7 days), 1 month (T3 = 3 × 31 days), 3 months (T4 = 3 × 90 days), 6 months (T5 = 3 × 181 days), 9 months (T6 = 3 × 273 days), and 12 months (T7 = 3 × 365 days) (the number of days refers to the cumulative period). This procedure was repeated 10 times for each attachment for all three platform inclination angles of 0, 10, and 20°. To further limit bias, the order that each attachment was tested in was randomized using a random sequence generator.

### 2.3. Data Analysis

To interpret the obtained data, mean retentive forces (N) were compared using general linear models, with the attachment type and time as fixed factors (α = 0.05). The effect of “groups” and “periods”, as well as their interaction, were evaluated. “Periods” were considered as a paired variable, different from “groups”. Further analysis was conducted using linear regression and respective 95% confidence intervals, as well as the Bonferroni test. Each analysis strategy was separately conducted for each of the inclination angles. Analyses were conducted using the SPSS software, v.23 (IBM SPSS Inc., Chicago, IL, USA), with α = 0.05.

## 3. Results

**Figure 2:**
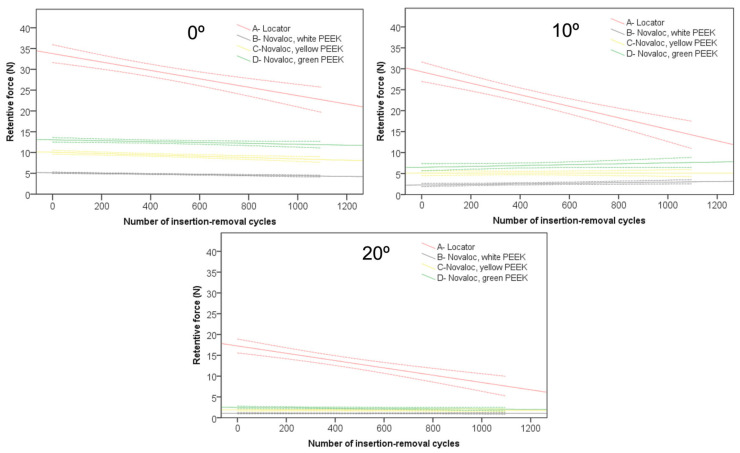
Retention forces of Locator and white, yellow, and green Novaloc attachments plotted against insertion–removal cycles at platform angles of 0°, 10°, and 20°.

Figure 2 and Table 1 present the mean retentive forces according to the removal cycles. The Locator attachments showed the highest initial retentive forces (36.0 ± 7.9, 35.3 ± 7.7, and 19.7 ± 5.5 N at 0°, 10°, and 20° inclines, respectively), while Novaloc attachments had the lowest retentive values (white: 5.8 ± 0.4, 2.4 ± 1.1, and 1.1 ± 0.2; yellow: 10.2 ± 0.8, 5.3 ± 1.6, and 1.7 ± 0.3; green: 13.1 ± 1.0, 7.2 ± 2.9, and 2.7 ± 1.0 N, at 0°, 10°, and 20° inclines, respectively). However, irrespective of the abutment angle (0°, 10°, and 20°), the Novaloc retentive components displayed the smallest losses in retentive forces with each removal–insertion cycle relative to its Locator counterpart.

From the linear regression analysis, taking into consideration all attachments together, the mean squares of the retentive forces across groups were highest at a 0° inclined abutment platform, followed by a 10° and 20° inclination (F _(3,6)_ = 177.278, *p* < 0.001, F _(3,6)_ = 269.970, *p* < 0.001, and F _(3,6)_ = 201.776, *p* < 0.001 at 0°, 10°, and 20° inclination, respectively; see Appendix A Table A1). Across periods (T1–T7), the retentive forces were largest with 10°, 0°, and 20°, respectively (F _(3,6)_ =10.354, *p* < 0.001, F _(3,6)_ = 15.874, *p* < 0.001, and F _(3,6)_ = 8.612, *p* < 0.001 corresponding to the mentioned angles; see Appendix A Table A1), suggesting that a 10° inclined platform resulted in a more pronounced retentive force loss over time. For all these results, there was a significant effect of insertion–removal cycles on retention forces (*p* < 0.05).

A negative beta value, given their *p* < 0.005 value, indicated that per each 1 unit increase in wear and tear cycles, there was a decrease in the retentive value by the determined beta value (see Appendix A Table A2). Amongst all retentive parts, the Locator system had the largest beta figure (B = 0.01 vs. −7.37 × 10^−4^, −1.6 × 10^−3^, and −1.07 × 10^−3^ for white, yellow, and green Novaloc, respectively, at a 0° incline), which further confirms the above-mentioned outcome that the largest retention force losses with the insertion–removal cycle are common to Locator. Across all inclination angles, the smallest beta value was within the white Novaloc attachment (B = −7.37 × 10^−4^, −6.98 × 10^−4^, and −2.72 × 10^−5^ at 0°, 10°, and 20° inclination, respectively). The highest r-squared values were within the Locator attachments, which solidifies the usefulness of the assigned beta figure.

After a statistically significant result being found in the ANOVA analysis, a post hoc procedure such as the Bonferroni test was utilized to compare each of the conditions (three retention forces of Novaloc plus and Locator (pink)) with one another (see Appendix A Table A3, Table A4 and Table A5).

At a 0°, 10°, and 20° platform inclination, the ANOVA yielded a significant group vs. retention interaction (*p* < 0.001). A post hoc test confirmed significant differences between Locator and all three Novaloc attachments (*p* = 0.000), where Locator indicated higher retention forces. Amongst the Novaloc attachments, the white attachment was statistically different when compared against yellow and green, and it showed statistically significant lower retentive forces (*p* = 0.000, see Table 1 and Appendix A Table A3) at a 0° and 10° platform inclination. At a 20° platform inclination, there was no statistically significant difference in retention forces amongst the three Novaloc attachment groups (white–yellow: t _3,6_ = −0.7866, *p* = 1.000, white–green: t _3,6_ = −1.2763, *p* = 0.236, and yellow–green: t _3,6_ = −0.490, *p* = 1.00) (Appendix A Table A5).

## 4. Discussion

This study elucidated the difference in retentive forces of the Novaloc attachment system relative to its traditional equivalent, the widely used Locator attachment (control). As indicated above, the results suggest that despite the highest retentive force values with the Locator attachment, the Novaloc system is more resistant to insertion–removal cycling; thus, it is likely more resistant to time-dependent retention loss. As such, this behavior should be taken into consideration by dentists when choosing both Novaloc and Locator attachments, as well as the long-term comfort of overdenture wearers, considering the time and the limited displacement of some denture wearers.

Our findings indicate that Locator attachments have the highest initial retention forces over Novaloc attachments. Irrespective of the implant analog platform inclination (0, 10, or 20°), all three Novaloc retentive components (PEEK white, yellow, and green) displayed the lowest loss in retention force with each removal–insertion cycle, which concurs with some studies [22,24]. While high retention forces are useful in ensuring the stability of a denture as observed with Locator, the rapid retentive loss can be noticeable to the patient and could explain a possible preference for a Novaloc attachment rather than Locator in some cases. This phenomenon was observed in a previous randomized cross-over trial by our group, with 7 out of 10 participants preferring Novaloc after 3 months of SIO wearing, in addition to significantly higher patient satisfaction [25]. Another study showed that patient satisfaction and perceived stability with implant overdentures are inversely proportional to retention loss with use, regardless of their initial retention [26]. The continuous retention force curve during the wearing period could be related to the preference of the majority of the patients for the Novaloc attachment in the study. However, we cannot discard the concept that Locator may be preferred by some patients, especially for shorter periods.

From the linear regression analysis, it was deduced that the highest retentive forces amongst all attachment groups were at a platform inclination of 0°, and the largest loss in retention across all groups was present at an incline of 10° followed by 0° and 20°. The present results indicate that the wear and tear of retentive components may not be affected by the parallelism of multiple implants or a perfectly linear path of insertion of a denture on an implant/fixture as previously believed [27,28,29,30]. However, 20° may be the limit for angulation. Studies have reported a substantial and considerable loss of retention when using angulations beyond 20° [22,24].

Moreover, an irrefutable finding is that the Locator attachment displays significantly higher retentive forces than all the Novaloc attachments (white, yellow, and green) at a 0° incline. Amongst Novaloc attachments, the yellow and green attachments display a significantly higher retention force relative to their white counterpart. This implies that the yellow and green attachments may perform better clinically, as they are more retentive and durable. The green Novaloc was observed to be marginally more performant (e.g., higher retention force) than the yellow Novaloc, which could be a clinical advantage. However, a retention of 5–7 N is considered enough to retain the overdentures [31].

Like the first experimental condition, the Locator attachment displayed significantly higher retentive forces then all the Novaloc attachments for both 10° and 20° inclines. At a 10° incline, the yellow and green attachments performed better (higher retention forces) than their white counterpart, whereas no statistical difference was found between the yellow and green Locator. At a 20° incline, no significant difference was found in the performance of any of the Novaloc attachments. What this indicates is that should an implant be placed at a deviation angle of 20° from the path of insertion, if a Novaloc is to be used, there are no added benefits in selecting one PEEK matrix over the other. Additionally, a slightly more pronounced retention loss over time was found with the 10° platform compared to the others. This was likely caused by more pronounced deformation with a mild incline compared to one of 0°. The retention loss was not so pronounced with 20°, most likely because this position does not provide firm engagement between the matrix and abutment undercuts.

The study demonstrates how much tested attachments can keep their retention force with insertion–removal cycling. However, the in vitro experimental design precludes the extrapolation of these results directly to clinical practice. Instead, these findings further explain the results obtained in our clinical trial, i.e., patients’ preference for Novaloc over Locator [25]. The present methods attempted to reproduce clinical conditions, including the insertion and removal of an SIO at different path of insertion angles. One may argue that using more sophisticated testing equipment would lead to less variation in results. However, the low standard deviations suggest that this is not the case and thus confirm the suitability of our test methods.

As a potential limitation, it is important to highlight that our study compared the Locator attachment with medium retention with three retention grades of Novaloc. Other retentive grades of both Locator and Novaloc could have been included and could have elicited different responses. However, a similar material composition would likely create a comparable resistance to cycling.

Using patients’ preference to determine the type of the retentive components chosen for an implant overdenture is an important approach. To help guide this decision, the results obtained from this study inform us about the deformation behavior or performance of PEEK and nylon matrices, which can more effectively assist dentists in counselling their patients. Should a higher initial retention force be favorable, the Locator attachment may be considered. If the patient favors more reliable and more progressive loss in the retention of the denture, a Novaloc attachment may be favored. The latter aspect may be critical in most SIO cases and may explain the selection of stronger PEEK matrices, as evidenced in our clinical trial [25]. To guarantee the generalization of these results, future practice-based studies and clinical trials should be carried out.

## Figures and Tables

**Table 1:** Mean (SD) values for each experimental condition. Roman numerals beside the groups represent results obtained via Bonferroni test for comparison regardless of cycling.

Angle	Groups *	T1, Baseline	T2, 21 Cycles	T3, 93 Cycles	T4, 270 Cycles	T5, 543 Cycles	T6, 819 Cycles	T7, 1095 Cycles
0°	A ^I^	36.0 (7.9)	31.7 (5.4)	32.1 (4.0)	31.1 (5.3)	28.3 (7.3)	26.1 (8.2)	22.3 (5.8)
	B ^II^	5.8 (0.4)	5.0 (0.5)	4.9 (0.4)	4.6 (0.5)	4.6 (0.3)	4.6 (0.3)	4.5 (0.4)
	C ^III^	10.2 (0.8)	9.5 (0.8)	9.7 (1.1)	10.3 (2.6)	9.4 (1.6)	8.6 (0.8)	8.2 (0.9)
	D ^III^	13.1 (1.0)	12.7 (1.7)	13.2 (2.4)	13.1 (2.0)	12.0 (1.3)	12.3 (1.6)	11.9 (1.2)
10°	A ^I^	35.3 (7.7)	29.9 (8.0)	22.9 (5.9)	22.6 (5.9)	22.3 (5.5)	17.0 (5.6)	15.9 (3.1)
	B ^II^	2.4 (1.1)	2.3 (0.9)	2.1 (0.8)	2.7 (1.1)	2.5 (1.2)	3.0 (1.2)	2.9 (1.3)
	C ^III^	5.3 (1.6)	4.9 (1.7)	4.9 (1.5)	5.4 (1.5)	5.3 (1.5)	5.7 (2.7)	4.6 (2.1)
	D ^III^	7.2 (2.9)	6.4 (2.5)	6.0 (2.4)	6.3 (2.8)	7.7 (3.1)	7.3 (2.1)	7.5 (2.0)
20°	A ^I^	19.7 (5.5)	15.0 (4.2)	16.3 (6.7)	15.0 (5.4)	11.1 (5.3)	11.3 (3.5)	7.4 (2.3)
	B ^II^	1.1 (0.2)	1.1 (0.3)	1.0 (0.4)	1.1 (0.4)	1.1 (0.4)	1.1 (0.5)	0.9 (0.3)
	C ^II^	1.7 (0.3)	1.9 (0.6)	2.0 (0.8)	2.0 (0.9)	1.6 (0.6)	2.0 (1.3)	1.7 (0.8)
	D ^II^	2.7 (1.0)	2.5 (1.2)	2.5 (1.0)	2.0 (0.4)	2.2 (0.9)	2.6 (0.9)	1.9 (0.4)

* A: Locator; B: Novaloc, white PEEK insert; C: Novaloc, yellow PEEK insert; D: Novaloc, green PEEK insert.

## Data Availability

The data presented in this study are available on request from the corresponding author.

## Appendix A

**Table A1:** General linear model output.

Angle	Source	df	Mean Square	F	*p*-Value *
0°	Groups	3	8222.981	177.278	<0.001
	Periods	6	91.076	15.874	<0.001
	Interaction	18	40.727	7.098	<0.001
10°	Groups	3	6439.278	269.970	<0.001
	Periods	6	106.863	10.354	<0.001
	Interaction	18	123.836	11.998	<0.001
20°	Groups	3	2516.817	201.776	<0.001
	Periods	6	45.893	8.612	<0.001
	Interaction	18	39.731	7.456	<0.001

* All *p*-values are significant (<0.05).

**Table A2:** Linear regression analysis.

Angle	Groups *	Intercept	Beta	Beta/100 Cycles	R^2^
0°	A	33.78	−0.01	−1	0.293
	B	5.14	−7.37 × 10^−4^		0.065
	C	10.07	−1.6 × 10^−3^		0.177
	D	13.06	−1.07 × 10^−3^		0.259
10°	A	29.29	−0.01	−1	0.396
	B	2.28	−6.98 × 10^−4^		0.027
	C	5.15	−2.51 × 10^−5^		3.126 × 10^−5^
	D	6.51	1.04 × 10^−3^		0.064
20°	A	17.26	−8.7 × 10^−3^		0.340
	B	1.07	−2.72 × 10^−5^		8.711 × 10^−4^
	C	1.86	−4.87 × 10^−5^		6.1 × 10^−4^
	D	2.49	−3.81 × 10^−4^		0.029

* A: Locator; B: Novaloc, white PEEK insert; C: Novaloc, yellow PEEK insert; D: Novaloc, green PEEK insert.

**Table A3:** Pairwise comparison at a 0° inclined platform.

Pairwise Comparisons						
Measure: Retention_N						
(I) Group	(J) Group	Mean Difference (I–J)	Std. Error	Sig.^a^	95% Confidence Interval for Difference ^a^	
					Lower Bound	Upper Bound
A—Locator	B—Novaloc w. white attachments	24.836 *	1.151	0.000	21.622	28.050
	C—Novaloc w. yellow attachments	20.255 *	1.151	0.000	17.041	23.469
	D—Novaloc w. green attachments	17.051 *	1.151	0.000	13.837	20.265
B—Novaloc w. white attachments	A—Locator	−24.836 *	1.151	0.000	−28.050	−21.622
	C—Novaloc w. yellow attachments	−4.581 *	1.151	0.002	−7.795	−1.367
	D—Novaloc w. green attachments	−7.785 *	1.151	0.000	−10.999	−4.571
C—Novaloc w. yellow attachments	A—Locator	−20.255 *	1.151	0.000	−23.469	−17.041
	B—Novaloc w. white attachments	4.581 *	1.151	0.002	1.367	7.795
	D—Novaloc w. green attachments	−3.204	1.151	0.051	−6.418	0.010
D—Novaloc w. green attachments	A—Locator	−17.051 *	1.151	0.000	−20.265	−13.837
	B—Novaloc w. white attachments	7.785 *	1.151	0.000	4.571	10.999
	C—Novaloc w. yellow attachments	3.204	1.151	0.051	−0.010	6.418

Based on estimated marginal means. * The mean difference is significant at the 0.05 level. a. Adjustment for multiple comparisons: Bonferroni.

**Table A4:** Pairwise comparison at a 10° inclined platform.

Multiple Comparisons						
Measure: Resistance_10°						
Bonferroni						
(I) Group	(J) Group	Mean Difference (I–J)	Std. Error	Sig.	95% Confidence Interval	
					Lower Bound	Upper Bound
A—Locator	B—Novaloc w. white attachments	21.1590 *	0.82552	0.000	18.8542	23.4638
	C—Novaloc w. yellow attachments	18.5840 *	0.82552	0.000	16.2792	20.8888
	D—Novaloc w. green attachments	16.7884 *	0.82552	0.000	14.4836	19.0933
B—Novaloc w. white attachments	A—Locator	−21.1590 *	0.82552	0.000	−23.4638	−18.8542
	C—Novaloc w. yellow attachments	−2.5750 *	0.82552	0.021	−4.8798	−0.2702
	D—Novaloc w. green attachments	−4.3706 *	0.82552	0.000	−6.6754	−2.0657
C—Novaloc w. yellow attachments	A—Locator	−18.5840 *	0.82552	0.000	−20.8888	−16.2792
	B—Novaloc w. white attachments	2.5750 *	0.82552	0.021	0.2702	4.8798
	D—Novaloc w. green attachments	−1.7956	0.82552	0.218	−4.1004	0.5093
D—Novaloc w. green attachments	A—Locator	−16.7884 *	0.82552	0.000	−19.0933	−14.4836
	B—Novaloc w. white attachments	4.3706 *	0.82552	0.000	2.0657	6.6754
	C—Novaloc w. yellow attachments	1.7956	0.82552	0.218	−0.5093	4.1004

Based on observed means. The error term is mean square (error) = 3.407. * The mean difference is significant at the 0.05 level.

**Table A5:** Pairwise comparison at a 20° inclined platform.

Multiple Comparisons						
Measure: Resistance_20°						
Bonferroni						
(I) Group	(J) Group	Mean Difference (I–J)	Std. Error	Sig.	95% Confidence Interval	
					Lower Bound	Upper Bound
A—Locator	B—Novaloc w. white attachments	12.6339 *	0.59698	0.000	10.9671	14.3006
	C—Novaloc w. yellow attachments	11.8473 *	0.59698	0.000	10.1805	13.5140
	D—Novaloc w. green attachments	11.3576 *	0.59698	0.000	9.6908	13.0243
B—Novaloc w. white attachments	A—Locator	−12.6339 *	0.59698	0.000	−14.3006	−10.9671
	C—Novaloc w. yellow attachments	−0.7866	0.59698	1.000	−2.4533	0.8802
	D—Novaloc w. green attachments	−1.2763	0.59698	0.236	−2.9430	0.3905
C—Novaloc w. yellow attachments	A—Locator	−11.8473 *	0.59698	0.000	−13.5140	−10.1805
	B—Novaloc w. white attachments	0.7866	0.59698	1.000	−0.8802	2.4533
	D—Novaloc w. green attachments	−0.4897	0.59698	1.000	−2.1565	1.1770
D—Novaloc w. green attachments	A—Locator	−11.3576 *	0.59698	0.000	−13.0243	−9.6908
	B—Novaloc w. white attachments	1.2763	0.59698	0.236	−0.3905	2.9430
	C—Novaloc w. yellow attachments	0.4897	0.59698	1.000	−1.1770	2.1565

The error term is mean square (error) = 1.782. * The mean difference is significant at the 0.05 level. Based on observed means.
